# The functional mechanism of miR-125b in gastric cancer and its effect on the chemosensitivity of cisplatin

**DOI:** 10.18632/oncotarget.23249

**Published:** 2017-12-14

**Authors:** Xinyue Zhang, Jie Yao, Kai Guo, Hu Huang, Siyuan Huai, Rui Ye, Baolong Niu, Tiannan Ji, Weidong Han, Jianxiong Li

**Affiliations:** ^1^ Department of Radiotherapy, Chinese PLA General Hospital, Beijing 100853, P.R. China; ^2^ Center for Evidence-Based and Translational Medicine, Zhongnan Hospital of Wuhan University, Wuhan 430071, P.R. China; ^3^ Department of Gastroenterology, The 161th Hospital of PLA, Wuhan 430010, P.R. China; ^4^ Department of Oncology, The 161th Hospital of PLA, Wuhan 430010, P.R. China; ^5^ Department of Oncology, Beidaihe Sanatorium of Beijing Military Command, Qinhuangdao 066100, P.R. China; ^6^ Department of Molecular Biology, Institute of Basic Medicine, School of Life Sciences, Chinese PLA General Hospital, Beijing 100853, P.R. China; ^7^ Department of Radiotherapy, Hainan Branch of Chinese PLA General Hospital, Sanya 572000, P.R. China

**Keywords:** miR-125b, HER2, gastric cancer, cisplatin, chemosensitivity

## Abstract

Numerous studies have shown drug resistance of gastric cancer cells could be modulated by abnormal expression of microRNAs. Cisplatin (DDP) is one of the most commonly used drugs for chemotherapy of gastric cancer. In this study, the potential function of miR-125b on DDP resistance in gastric cancer cells was investigated. Sixteen miRNAs significantly differential expressed in gastric tumor tissues and adjacent tissues were characterized and their corresponding putative target genes were also screened. MiR-125b was selected as our focus for its evident down-regulated expression among candidate genes. Real-time polymerase chain reaction assay indicated that miR-125b was significantly down-regulated in gastric cancer tissues and various cell lines. HER2 was identified as a target gene of miR-125b by dual luciferase reporter assay and Western blot. Moreover, miR-125b overexpression inhibited not only the proliferation, migration, and invasion abilities of HGC-27 and MGC-803 cells, but also *in vivo* tumor growth of MGC-803 cells by an intratumoral delivery approach. Notably, we observed up-regulated miR-125b contributed to the chemosensitivity of DDP in HGC-27 and MGC-803 cells at different concentrations and also possessed sensibilization for DDP at different times. MiR-125b expression was found to be related to lymph node metastasis, HER2 expression and overall survival of patients through correlation analysis. Collectively, these results indicate miR-125b may regulate DDP resistance as a promising therapeutic target for gastric cancer treatment in future.

## INTRODUCTION

Gastric cancer is one of the most common malignant diseases and the second leading cause of cancer-related deaths worldwide, especially in the Asian countries [1]. Despite great advances in surgical techniques and chemotherapeutic agents for gastric cancer, the 5-year-survival rate in patients is still unsatisfactory due to its metastasis and chemoresistance [2, 3]. Therefore, it is indispensable to identify potential novel chemotherapy targets and understand their molecular mechanisms for improving the prognosis of gastric cancer.

Mounting evidence demonstrates that microRNAs (miRNAs) are involved in the development and progression of tumors as oncogenes or tumor-suppressors [4]. miRNAs are a class of extremely important regulatory RNA molecules influencing gene function through inhibiting translation or inducing the degradation of target mRNA [5]. It has been widely reported that miRNAs play a vital role in regulating biological processes, including cell proliferation, migration, invasion, and apoptosis [6–8]. Importantly, abnormal expression caused by miRNA regulation could lead to the pathogenesis and development of various tumors [9]. For example, numerous studies indicate that miR-21 is implicated in colorectal cancer [10], hepatocellular carcinoma [11], gastric cancer [12] and other cancers. Another miRNA, miR-125b, has also been shown to exert abnormal expression in a variety of tumors and its effects vary with different tumors [13, 14]. In gastric cancer, miR-125b overexpression may increase proliferation but decrease apoptosis [15]. However, in breast cancer, miR-125b can significantly inhibit tumor proliferation [16]. Additionally, the significant effect of miR-125b on the response to radiochemotherapy in various cancers, such as glioblastoma [17], oral squamous cell carcinoma [18] and ovarian cancer [19], has also been proved. However, the mechanism of miR-125b and its impact on the chemosensitivity in gastric cancer remains largely unknown.

Human epidermal growth factor receptor 2, also known as HER2, ERBB2 or c-erbB-2, is a proto-oncogene that encodes a trans-membrane receptor with a constitutive tyrosine kinase activity involved in cellular proliferation, differentiation, migration and apoptosis [20]. Emerging evidence has demonstrated that HER2 plays a critical role in tumorigenesis and neoplastic development [21, 22]. HER2 as a prognostic and selective biomarker in gastric cancer has aroused great attention [23, 24].

In this study, the significantly differential expressed miRNAs and the putative target genes were primarily characterized using microarray analysis and prediction software (Targetscan). We investigated the functional mechanism of miR-125b and its effect on the chemosensitivity of DDP in gastric cancer. Dual luciferase reporter assay and Western blot assay were conducted to verify whether HER2 was a direct target gene of miR-125b. An intratumoral delivery approach was conducted to investigate the influence of miR-125b on the *in vivo* tumor growth of MGC-803 cells. In addition, the correlation between miR-125b and clinical indicators was analyzed. The chemosensitivity effect of miR-125b on the DDP was also explored. Our results reveal that miR-125b plays a crucial role in gastric cancer which may lay a foundation for its application in clinical diagnosis and treatment.

## RESULTS

### Chip scanning and target prediction

**Figure 1:**
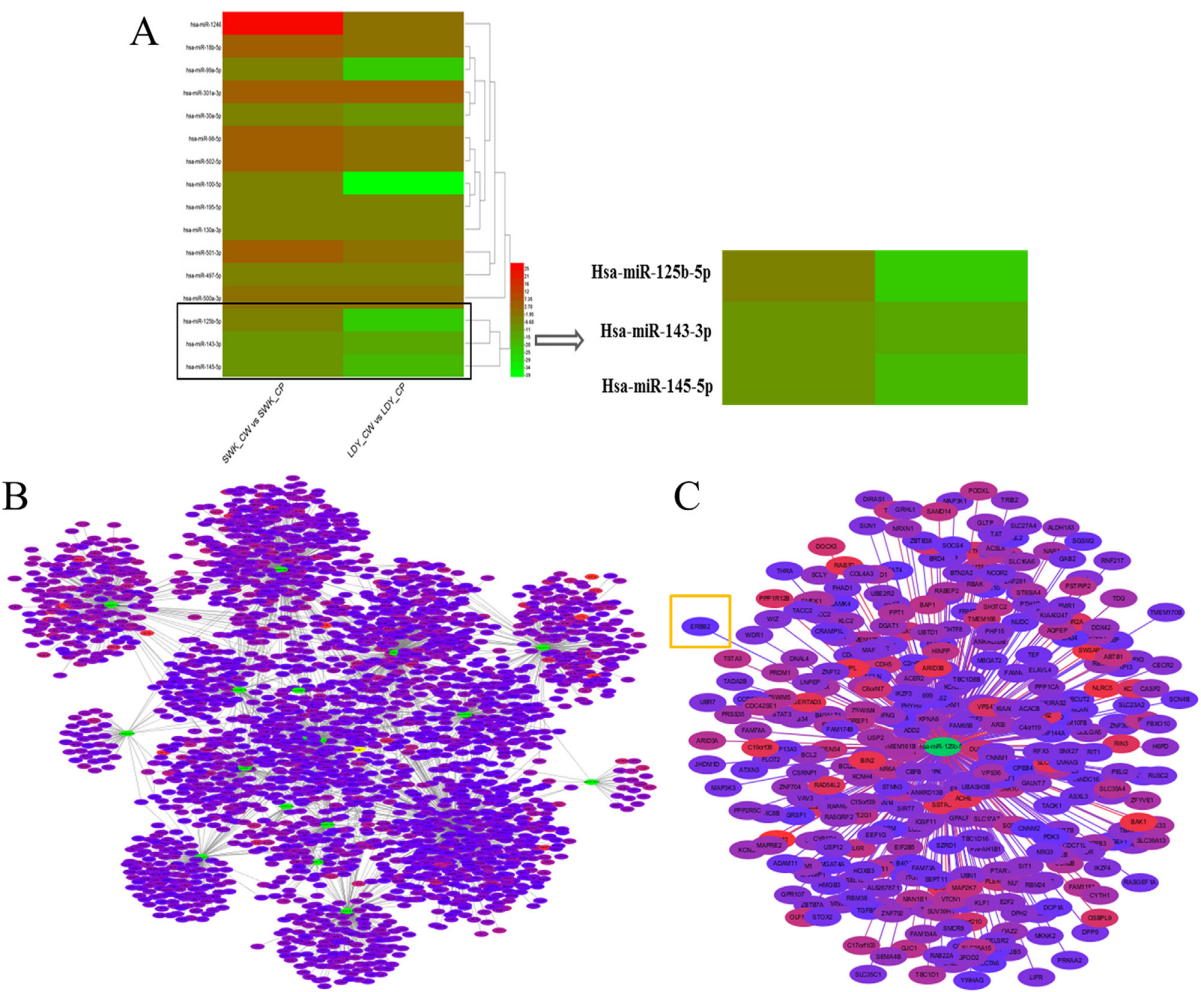
Results of chip scanning and target prediction of miRNAs in gastric cancer tissues **(A)** Heat maps of significantly differentially expressed miRNAs in gastric cancer tissues via microarray analysis. LDY_CP, LDY_CW, SWK_CP and SWK_CW are all sample IDs. Among them, CP represents adjacent normal tissues and CW refers to gastric cancer tissues. Red represents higher expression and green stands for lower expression. **(B)** Network of the putative target genes of significantly differentially expressed miRNAs according to Targetscan. Selected criteria was the cumulative weighted context++ score < -0.1. The green stands for miRNA and other colors represent the putative target genes. The more red the color presents, the higher the reliability exerts. **(C)** The enlarged network of the putative target genes of miR-125b.

According to microarray analysis, we had characterized sixteen miRNAs significantly differential expressed in tumor tissues and adjacent tissues based on fold change more than 2. Among these candidate miRNAs, miR-125b was selected as our target miRNA for the fact that its expression was evidently down-regulated (Figure 1A). Commonly, the biological functions of miRNAs were determined by their downstream target genes. Hence, the prediction software (Targetscan) was employed to primarily screen the putative target genes of those significantly differentially expressed miRNAs based on the cumulative weighted context++ score < -0.1 (Figure 1B). The putative target genes of our target miRNA were displayed in Figure 1C.

### miR-125b was down-regulated in gastric cancer samples compared with adjacent normal tissues

**Figure 2:**
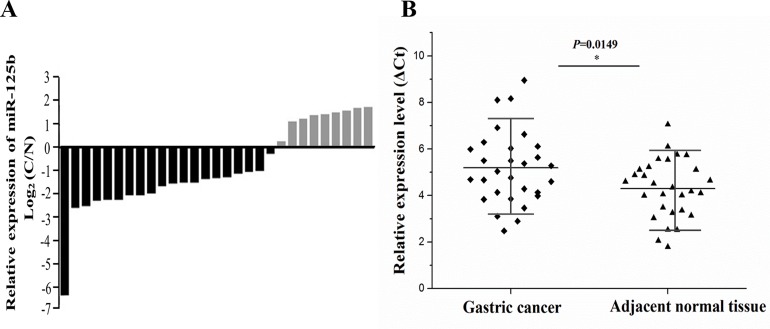
The expression of miR-125b was measured in 29 cases of gastric cancer and adjacent normal tissues **(A)** The miR-125b expression level was assessed in a panel paired specimen using RT-qPCR. **(B)** The statistical analysis of miR-125b relative expression level in tumor tissues and adjacent tissues using T-test. Horizontal line stands for the average value for each group. The ordinate represents ΔCt values of miR-125b expression. In general, higher ΔCt value stands for lower expression of miRNA. RT-qPCR, real-time quantitative polymerase chain reaction. Ct, cycle threshold; ^*^, *P* < 0.05.

The miR-125b expression level was assessed in a panel paired specimen obtained from 29 patients with gastric cancer. The data showed that miR-125b was mostly down-regulated in the gastric cancer tissues compared with matched normal tissues, accounting for 68.7% (Figure 2A). T-test was used to analyze the difference between gastric cancer tissues and matched normal tissues according to their corresponding ΔCt values of miR-125b expression. In general, higher ΔCt value stands for lower expression of miRNA. As depicted in Figure 2B, a clear-cut distinction was drawn between gastric cancer group and normal group, revealing miR-125b was significantly down-regulated in gastric cancer group.

### miR-125b was down-regulated in gastric cancer cell lines

**Figure 3:**
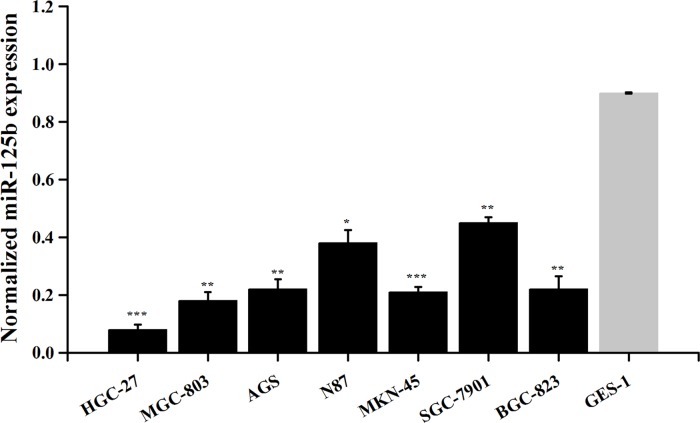
Expression of miR-125b in various gastric cancer cell lines Error bars symbolized standard deviation acquired from three independent experiments and all the data were shown as mean ± SD. ^*^, *P* < 0.05; ^**^, *P* < 0.01; ^***^, *P* < 0.001.

The expression levels of miR-125b in gastric cancer cell lines HGC-27, MGC-803, AGS, N87, MKN-45, SGC-7901, BGC-823 and normal cell line GES-1 were observed. The results revealed that the expression levels of miR-125b were significantly lower in gastric cancer cell lines than those in normal cell line (Figure 3, *P* < 0.05).

### HER2 was a direct target of miR-125b

**Figure 4:**
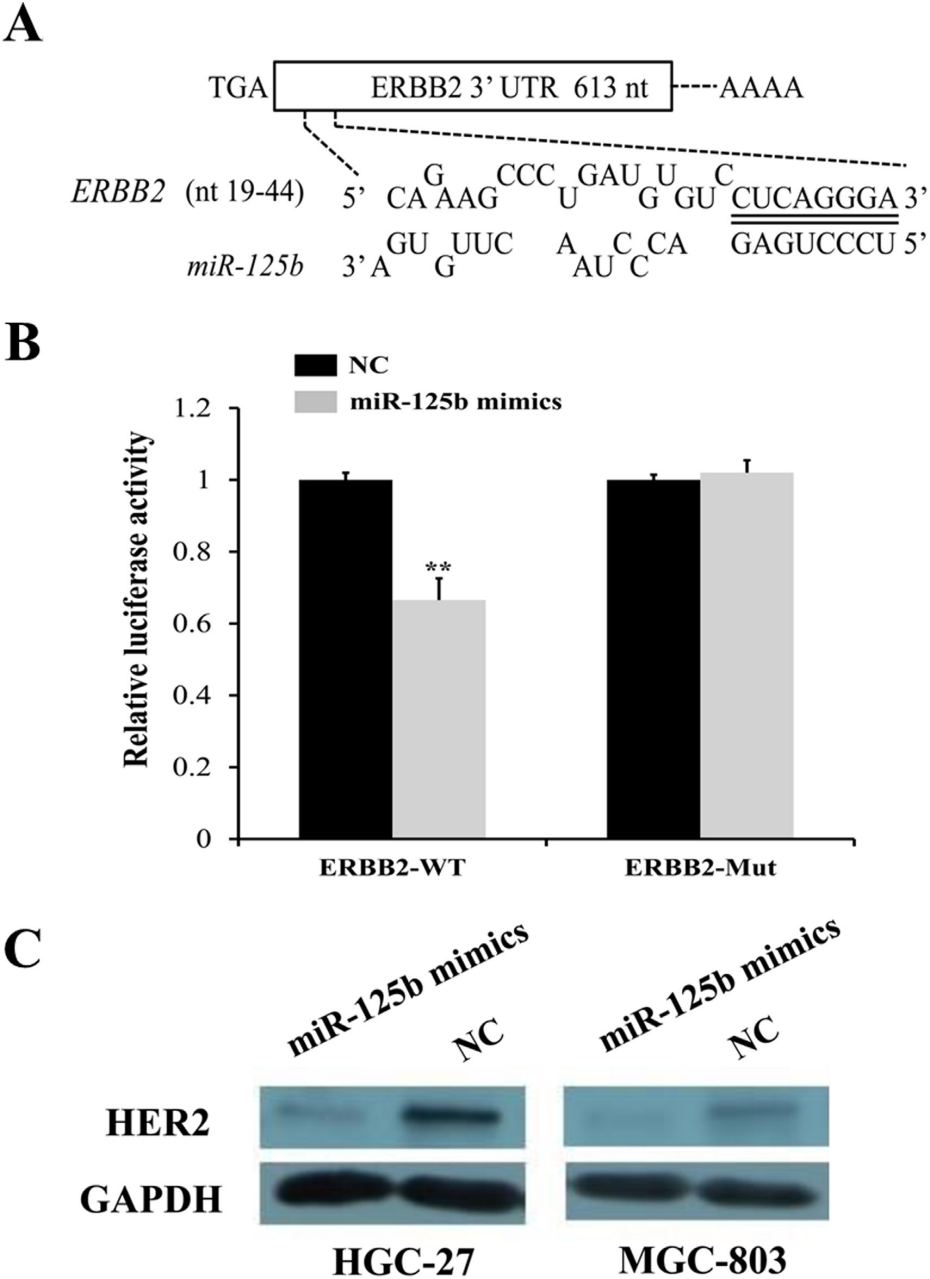
HER2 was identified as a direct target of miR-125b by target gene prediction software, dual luciferase reporter assay, and Western blot **(A)** Three target prediction databases, TargetScan, miRanda and PicTar were conjunctively used. There existed one predicted binding site in miR-125b matching well with the 3'-UTR of ERBB2 (also namely HER2). **(B)** Two luciferase reporter vectors that contained the putative miR-125b binding sites within HER2 3’UTR and mutant 3’UTR in 293TN cells were performed. **(C)** The protein level of HER2 in the HGC-27 and MGC-803 transfected with miR-125b mimics and NC was analyzed by Western blot. Error bars symbolized standard deviation were obtained from three independent experiments and all the data were shown as mean ± SD. NC, negative control; nt, nucleotide; WT, wild type; Mut, mutant type; GAPDH, glyceraldehyde-3-phosphate dehydrogenase; ^*^, *P* < 0.05; ^**^, *P* < 0.01.

To further explore the candidate target genes of miR-125b in gastric cancer, three target prediction databases, TargetScan (http://www.targetscan.org/), miRanda (http://www.microrna.org/), and PicTar (http://pictar.mdc-berlin.de/) were conjunctively used. As described in Figure 4A, there existed one predicted binding site in miR-125b matching well with the 3'-UTR of ERBB2 (also namely HER2), which had been identified as oncogene and also presented in Figure 1C (highlighted in orange box). To further confirm this observation, we conducted dual luciferase reporter vectors that contained the putative miR-125b binding sites within HER2 3’-UTR and mutant 3’-UTR in 293TN cells. The results uncovered that the relative luciferase activity was significantly decreased in the putative miR-125b mimics treated with wild-type HER2 3’-UTR group compared with NC group. In contrast, the luciferase activity in cells controlled by mutant HER2 3’-UTR almost was paralleled with that controlled by NC cells, indicating that HER2 was a target of miR-125b in gastric cancer cells (Figure 4B, *P* < 0.01). In addition, the protein level of HER2 in the HGC-27 and MGC-803 transfected with miR-125b mimics and NC was analyzed by Western blot. We found that miR-125b obviously inhibited the expression of HER2 at protein level in HGC-27 and MGC-803 cells (Figure 4C). Just as mentioned above, all results suggested that HER2 was a direct target of miR-125b, which was in line with the conclusion of Scott GK [25].

### Overexpression of miR-125b suppressed the proliferation, migration and invasion abilities of HGC-27 and MGC-803 cells

**Figure 5:**
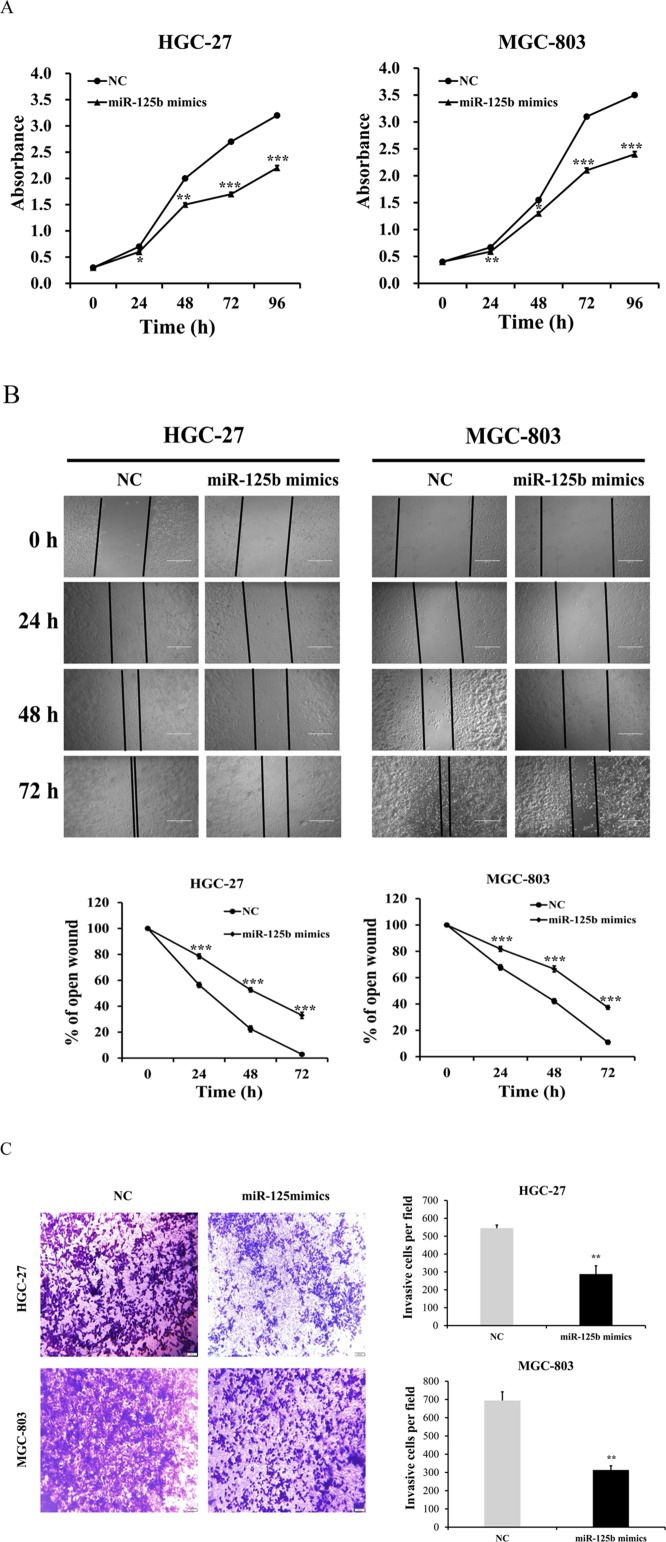
miR-125b expression levels inhibited the proliferation, migration and invasion abilities of HGC-27 and MGC-803 cells **(A)** In miR-125b overexpression experiment, the data indicated that both the proliferation of HGC-27 and MGC-803 cells in miR-125b mimics groups were evidently retarded compared with control group. **(B)** The wound-healing assay showed that HGC-27 and MGC-803 cells migration in miR-125b mimics groups were inhibited compared with the control groups. **(C)** The matrigel invasion assay also displayed the suppression in miR-125b mimics groups compared with the control groups. Error bars symbolized standard deviation were obtained from three independent experiments and all the data were shown as mean ± SD. All scale bars in Figure B and Figure C are 200 μm and 500 μm, respectively. NC, negative control; h, hour; ^*^, *P* < 0.05; ^**^, *P* < 0.01; ^***^, *P* < 0.001.

To further identify the role of miR-125b in HGC-27 and MGC-803 cells, functional assay was performed by transfecting miR-125b mimics and negative control (NC) group into cells. In the miR-125b overexpression experiment, the data indicated that the proliferation abilities of HGC-27 and MGC-803 cells in miR-125b mimics groups were evidently retarded compared with control group (Figure 5A, *P* < 0.05). The wound-healing assay showed that the migration abilities of HGC-27 and MGC-803 cells in miR-125b mimics groups were inhibited compared with the control groups (Figure 5B, *P* < 0.001). Furthermore, the matrigel invasion assay also displayed the suppression in miR-125b mimics groups compared with the control groups (Figure 5C, *P* < 0.01). All these results demonstrated that miR-125b could be the tumor-inhibiting factor which might obviously suppress the proliferation, migration and invasion abilities of HGC-27 and MGC-803 cells.

### Overexpression of miR-125b markedly inhibited *in vivo* tumor growth of MGC-803 cells

**Figure 6:**
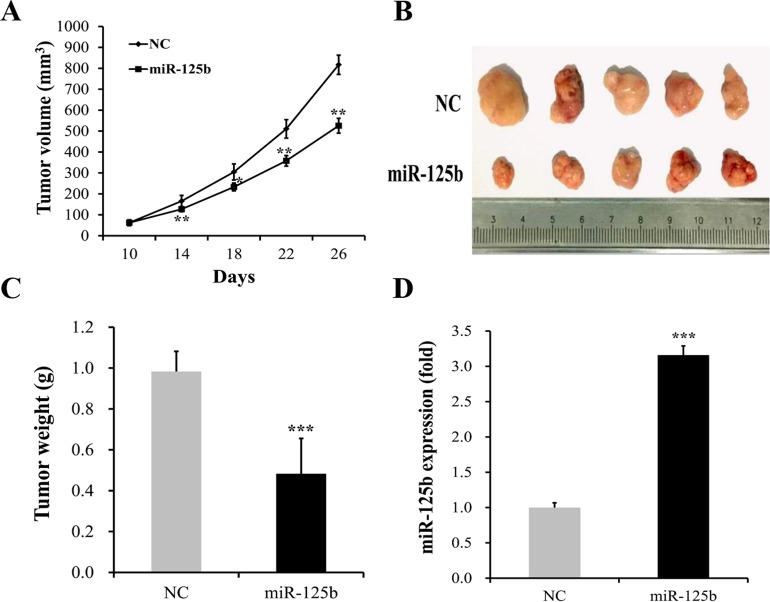
miR-125b overexpression reduced tumor growth of MGC-803 cells in vivo **(A)** The growth of tumor volume in miR-125b transfected group was significantly slower than that in NC group. **(B)** The tumor volumes in miR-125b transfected group were evidently smaller than those in NC group. **(C)** The tumor weight evidently increased in NC group when compared with miR-125b-treated group. **(D)** Real-time polymerase chain reaction (Real-time PCR) assay showed that miR-125b expression in xenograft tumors in the miR-125b transfected group was significantly increased compared with NC group. Error bars symbolized standard deviation were obtained from three independent experiments and all the data were shown as mean ± SD. NC, negative control; ^*^, *P* < 0.05; ^**^, *P* < 0.01; ^***^, *P* < 0.001.

To further determine the effect of miR-125b overexpression on gastric cancer progression *in vivo*, an intratumoral delivery approach was conducted in nude mice. We found that the growth of tumor volume in miR-125b transfected group was significantly slower than that in NC group (Figure 6A). At the same time, we also observed that the tumor volumes in miR-125b transfected group were evidently smaller than those in NC group (Figure 6B). The tumor weight evidently increased in NC group when compared with miR-125b mimics group (Figure 6C, *P < 0.05*). In addition, real-time PCR assay showed that miR-125b expression in xenograft tumors in the miR-125b transfected group was significantly increased compared with NC group (Figure 6D, *P < 0.05*)

### Overexpression of miR-125b improved the chemosensitivity of DDP in HGC-27 and MGC-803 cells

**Figure 7:**
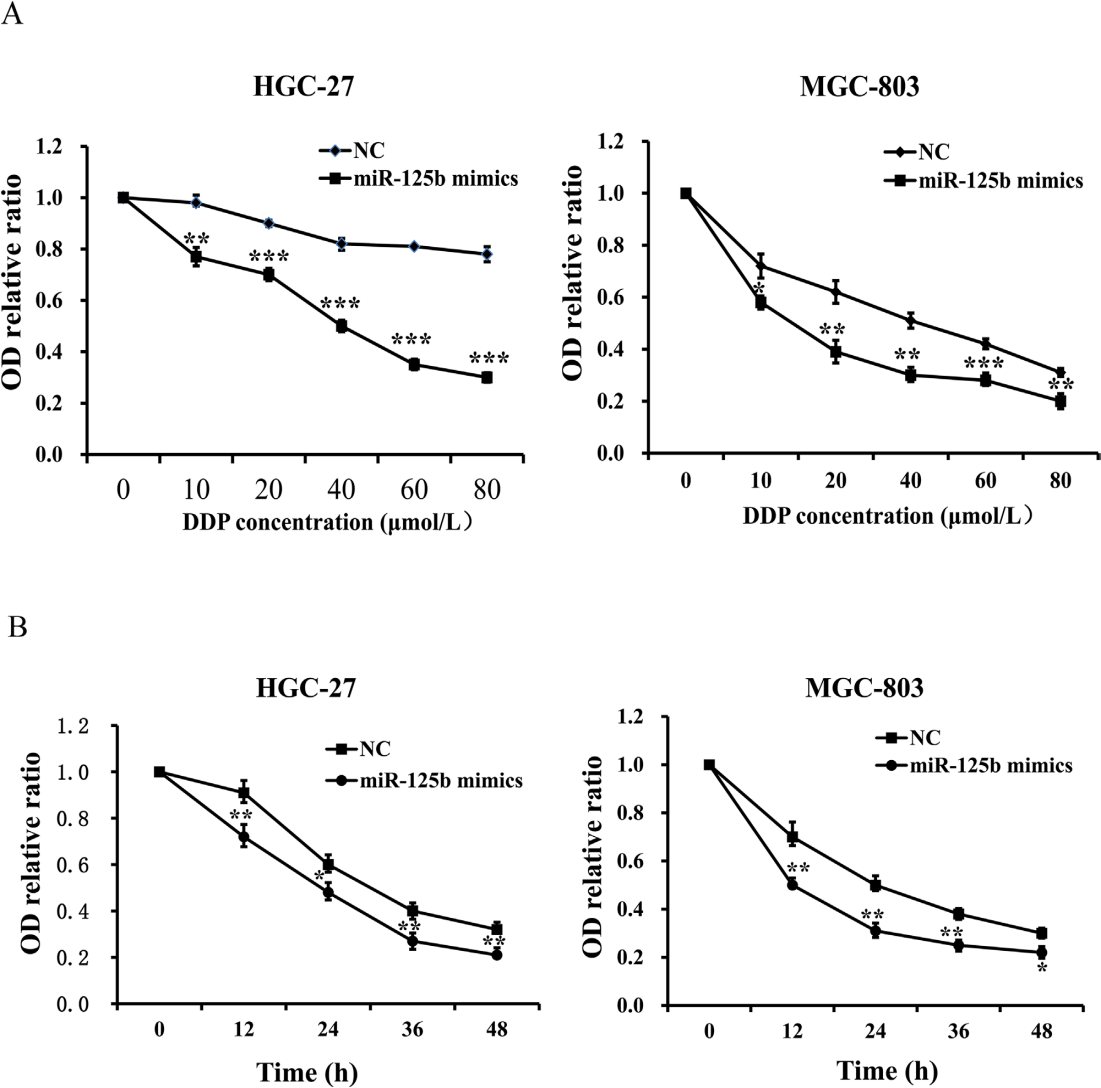
Overexpression of miR-125b contributed to the chemosensitivity of DDP in HGC-27 and MGC-803 cells **(A)** The OD levels of HGC-27 and MGC-803 cells with different concentration of DDP in miR-125b mimics groups were evidently lower than those in the control groups. **(B)** The OD levels of HGC-27 and MGC-803 cells in miR-125b mimics groups at different times were evidently lower than those in the control groups. Error bars symbolized standard deviation were obtained from three independent experiments and all the data were shown as mean ± SD. NC, negative control; DDP, cisplatin; OD, optical density; ^*^, *P* < 0.05; ^**^, *P* < 0.01; ^***^, *P* < 0.001.

To determine the influence of DDP concentration on the chemosensitivity of miR-125b, HGC-27 and MGC-803 cells were treated with miR-125b mimics and miR-125b control group, respectively. Different concentrations of DDP (0, 10, 20, 40, 60 and 80 μmol/L) were added into each group when cells became adherent. Afterwards, the viability of those two cells treated with different concentrations of DDP was evaluated by CCK-8 assay. We found that the OD levels of HGC-27 and MGC-803 cells with different concentrations of DDP in miR-125b mimics groups were evidently lower than those in the control groups (Figure 7A, *P* < 0.05). The results revealed that miR-125b overexpression contributed to the chemosensitivity of DDP in HGC-27 and MGC-803 cells at different concentrations of DDP, and identified that the IC50 value of DDP in gastric cancer cell lines HGC-27 and MGC-803 was about 50 μmol/L, which laid the foundation for the next study. To further explore the influence of functional time on the chemosensitivity improvement of miR-125b for DDP in gastric cancer cells, CCK-8 assays were conducted after adding 50 μmol/L (IC50) DDP for 0, 12, 24, 36 and 48 h, respectively. We also observed that the OD levels of HGC-27 and MGC-803 cells in miR-125b mimics groups at different times were evidently lower than those in the control groups (Figure 7B, *P* < 0.05). The findings exhibited that miR-125b overexpression could improve the chemosensitivity of DDP in HGC-27 and MGC-803 cells at different times.

### The correlation analysis between miR-125b expression and clinical manifestation and prognosis of gastric cancer patients

**Figure 8:**
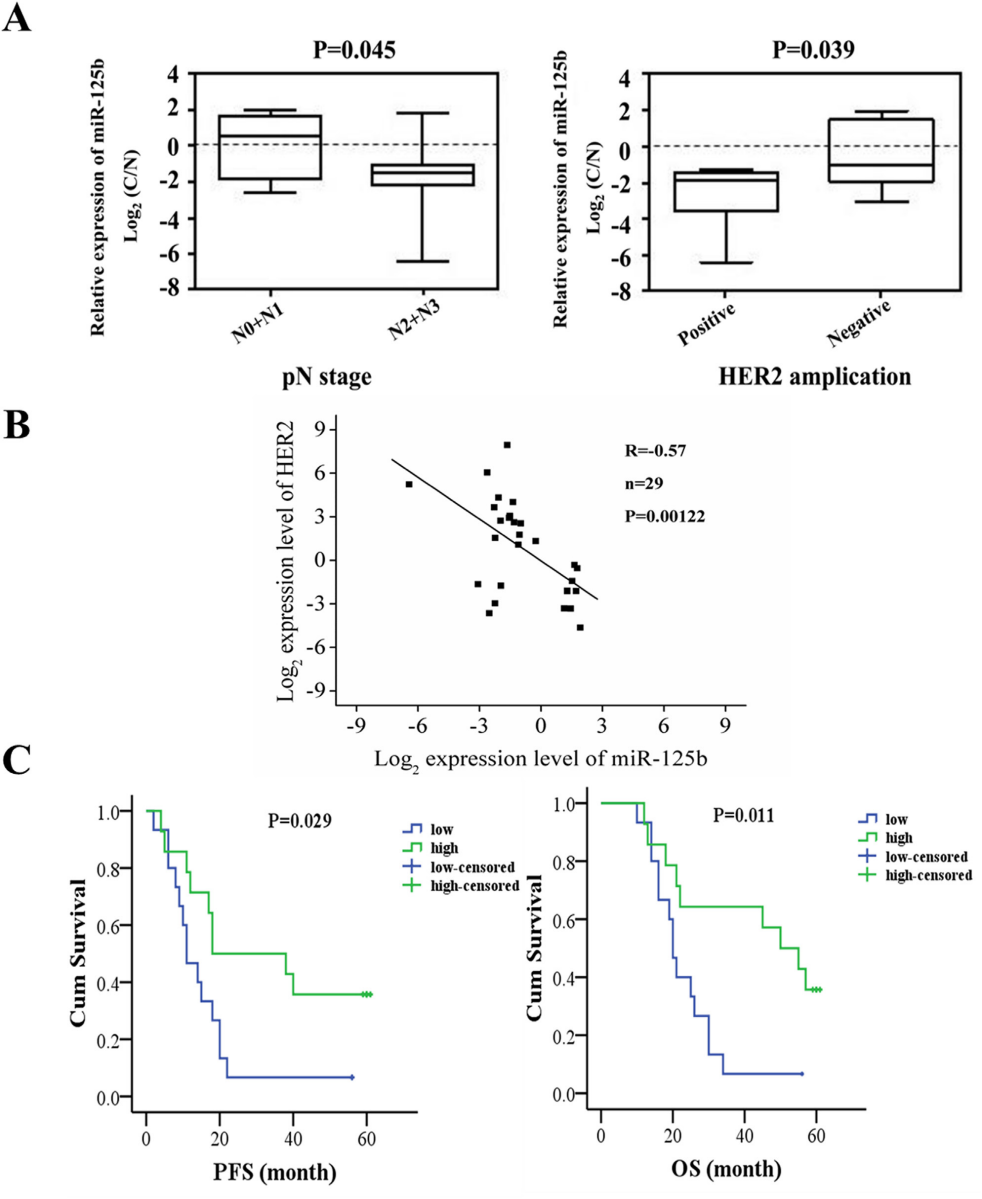
The correlation analysis between miR-125b expression and clinical manifestation and prognosis of gastric cancer patients **(A)** The expression level of miR-125b was associated with gender, lymph node metastasis and HER2 expression. **(B)** The correlation plot between miR-125b expression levels and HER2 expression. **(C)** PFS and OS of gastric cancer patients were analyzed in miR-125b high expression and miR-125b low expression, respectively. The patients were stratified by the median value of the expression found in all the samples. Error bars symbolized standard deviation were obtained from three independent experiments and all the data were shown as mean ± SD. C: normalized expression of cancer tissues; N: normalized expression of adjacent noncancerous tissues; PFS, progression free survival; OS, overall survival.

The clinical data of the 29 patients are presented in Table 1. We observed that the expression level of miR-125b was associated with gender, lymph node metastasis and HER2 expression (Figure 8A, *P* < 0.05). The correlation plot between miR-125b expression levels and HER2 expression in 29 patients was also presented in Figure 8B, indicating the negative correlation of miR-125b expression levels and HER2 expression (R=-0.57, P=0.00122). However, the miR-125b expression had no significant correlation with other clinicopathological features such as age, tumor location, lymphovascular invasion, nerve invasion, Borrmann type and TNM stage and grade.

**Table 1:** Correlation analysis between miR-125b expression and clinical indicators of patients

Clinicopathological characteristics	Number of cases	mean^1^	SD^2^	P-value
Gender				0.044
Male	22	-1.341	1.794	
Female	7	0.494	1.852	
Age				0.425
≤60	16	-0.628	1.819	
>60	13	-1.231	2.119	
Venous Invasion				0.968
Positive	15	-0.913	1.591	
Negative	14	-0.883	2.331	
Perineural Invasion				0.970
Positive	4	-0.932	1.792	
Negative	25	-0.893	2.005	
Tumor location				0.294
Cardia	7	-1.639	0.707	
Gastric Body	4	0.351	1.550	
Gastric Antrum	18	-0.888	2.246	
Borrmann stage				0.727
I+II	15	-0.772	1.755	
III+IV	14	-1.034	2.192	
pT stage				0.954
T1+T2+T3	6	-0.856	1.894	
T3+T4	23	-0.909	2.002	
pN stage				0.045^*^
N0+N1	12	-0.042	1.815	
N2+N3	17	-1.503	1.851	
pM stage				
M0	28	-0.971	1.942	
M1	1	1.131		
pTNM stage				0.511
I+II	6	-0.441	1.788	
III+IV	23	-1.018	2.006	
Histologic grade				0.282
Well	10	-1.501	2.283	
Poor	19	-0.581	1.726	
HER2 amplification				0.039^*^
Positive	6	-2.629	1.928	
Negative	23	-0.447	1.717	

1: mean of Log_2_ (C/N); 2: Standard Definition of Log_2_ (C/N); C: normalized expression of cancer tissues; N: normalized expression of adjacent noncancerous tissues.

The median expression level of miR-125b in all the tumor samples was defined as a cutoff to divide the patients into high expression and low expression groups. The progression free survival (PFS) and overall survival (OS) of gastric cancer patients were analyzed. The expression of miR-125b was significantly correlated with PFS, so was the case in the association with OS (Figure 8C, *P* < 0.05). In detail, the miR-125b high expression group was associated with a 35.7% postoperative survival rate, whereas only a 6.7% survival rate in the miR-125b low expression group. The results indicated that there existed significant differences between the two groups. The statistical analysis revealed that patients in high expression group had higher OS rate than those in low expression group, suggesting that miR-125b expression might be related to the prognosis of gastric cancer patients.

## DISCUSSION

MiRNA is a class of non-coding microRNA molecule with a length of about 22 nucleotides, which can regulate gene expression and plays important roles in physiologic and pathologic processes [7, 9]. Recent studies have revealed that miRNAs are involved in the regulation of cancer cells including their sensitivity to chemotherapy, such as miRNA-128 [26], miRNA-195 [27]. Aberrant expressions of miRNAs are closely linked with the occurrence and development of gastric cancer [9, 28]. In gastric cancer, miRNAs are also associated with cell signaling pathways, such as Ras / Raf / MEK / ERK, Wnt / β-Catenin / Tcf, Slit / Robo [29]. Moreover, some miRNAs have been reported to contribute to chemotherapy resistance, such as miR-503 [30] and miR-1271 [31]. Therefore, miRNA may be used as not only an important diagnosis biomarker for gastric cancer, but also an essential target in the treatment of gastric cancer.

Although the expression and functional role of miR-125b in different types of tumors are controversial, relevant studies show that it mostly acts as a tumor suppressor gene in solid tumors [32]. For example, miR-125b could inhibit invasion and metastasis of tumor cells, and it has been identified as a tumor suppressor gene in breast cancer [16] and bladder cancer [33]. Its expression levels in serum can be regarded as a biomarker to identify whether patients with non-small cell lung cancer could receive DDP-based chemotherapy [34]. However, biological mechanisms in the relationship between miR-125b and DDP chemotherapeutic resistance have not been fully investigated. Hence, this study was aimed to reveal the function of miR-125b in gastric cancer and explore its role in the chemotherapy resistance of DDP.

In this study, sixteen miRNAs significantly differential expressed in gastric cancer tissues and adjacent tissues were screened out and their corresponding putative target genes were also predicted. MiR-125b was selected as our focus for the fact that it was obviously down-regulated in the 29 gastric cancer samples through microarray analyses. This indicated that miR-125b might be a tumor suppressor in gastric cancer. The result of miR-125b targeting regulation of HER2 in gastric cancer cells was confirmed in our study by target gene prediction software, dual luciferase reporter assay, Western blot. Previous studies have shown that miR-125b directly or indirectly regulates HER2 in a great body of cancers. For example, the HER2 status (IHC and CISH) was reported to be correlated inversely with miR-125 expression in gastric and esophageal carcinogenesis. The reduced level of miR-125b contributed to the increased level of HER2, which promoted the cell proliferation and reduced the apoptotic rate in gastric and esophageal carcinogenesis [35]. MiR-125b expression could significantly down-regulate HER2 proteins in chondrosarcoma cells, and its knockdown up-regulated the expression of HER2 [36]. Another example is that the down-regulation of miR-125b resulted in up-regulation of HER2 in small cell lung cancer cells [37]. We also conducted a correlation between miR-125b expression levels and HER2 in 29 gastric cancer patients, suggesting the negative correlation of miR-125b expression levels and HER2 expression. A series of functional assays are subsequently performed, we identified that miR-125b could repress the proliferation, migration, and invasion abilities of gastric cancer cells *in vitro*. In order to further confirm the impact of miR-125b on gastric cancer progression *in vivo*, we used intratumoral delivery approach, which suggested that tumor volumes and weights significantly reduced after intratumoral delivery of miR-125b into subcutaneous xenografts. And miR-125b expression in the miR-125b transfected group was significantly higher than that in NC group, revealing the crucial role of miR-125b in tumor progression *in vivo*. More importantly, we found that overexpression of miR-125b improved the chemosensitivity of DDP both in HGC-27 and MGC-803 cells, which prolonged the PFS and OS of gastric cancer patients, underscoring the therapeutic potential of miR-125b in gastric cancer. We also observed that the expression level of miR-125b was associated with gender, lymph node metastasis and HER2 expression.

In conclusion, our study demonstrates that miR-125b is a tumor suppressor in gastric cancer. Overexpression of miR-125b results in the suppression of proliferation, migration and invasion abilities of HGC-27 and MGC-803 cells *in vitro* and tumor growth *in vivo*. We also affirm that HER2 is a direct target of miR-125b. Meantime, miR-125b overexpression could contribute to the chemosensitivity of DDP in gastric cancer cells and prolong the PFS and OS of gastric cancer patients. Despite of the research conducted as mentioned above, there still exist some limitations in our study. On the one hand, only a fraction of patient samples were analyzed in our study because of the difficulty in collection. We are planning to collect a great body of samples for analysis on no small measure. On the other hand, more detailed studies of the specified mechanism responsible for the biological functions of miR-125b in gastric cancer should be further performed, such as functional assay in gastric cancer cells not expressing miR-125b silenced for HER2, the correlation analysis between HER2 expression and prognosis of gastric cancer patients. We will follow up these directions to perform further investigations on the basis of this paper.

## MATERIALS AND METHODS

### Patient specimens

We obtained the specimens of tumors surgically removed from 29 patients who had diagnosed with gastric cancer. All patients had no therapy before surgery. All samples were preserved in liquid nitrogen at time of resection until analysis. Clinicopathological details of all patients including gender, age, venous invasion, perineural invasion, tumor location, Borrmann stage, pT stage, pN stage, pM stage, histologic grade and HER2 amplification. The status of HER2 (3+) was identified as positive and the status of HER2 (2+) was further verified by fluorescence *in situ* hybridization (FISH). The median value of miR-125b expression in patients was selected as the criterion for the definition of low miR-125b (< median value) and high miR-125b (> median value). Written informed consent was given in this study. The ethics approval for this research was granted by the Human Research Ethics Committee of Chinese PLA General Hospital.

### Cancer cell lines and cell culture

The human normal gastric mucosa epithelial cell lines GES-1, 293TN, and the human gastric cancer cell lines HGC-27, MGC-803, AGS, N87, MKN-45, SGC-7901, BGC-823 were obtained from Chinese Academy of Medical Science. GES-1, HGC-27, N87, MKN-45, SGC-7901, BGC-823 cells were cultured in RPMI-1640. 293TN, MGC-803 cells were cultured in DMEM (Dulbecco’s Modified Eagle’s Medium). AGS cells were cultured in F-12K (GIBCO). All the cell lines were supplemented with 10% fetal bovine serum(FBS) (GIBCO), 100 U/mL Penicillin (Sigma, USA) and 100 μg/mL Streptomycin (Sigma, USA) in the atmosphere of 37°C containing 5% CO_2_.

### Plasmid and transfection

MiRNA transfection was performed with Giemsa transfection reagent (AppliChem, Germany). In brief, cells were plated in each well of 12-well plates at least 24 h prior to transfection in order to achieve 50-70% confluency. MiRNA transfection was then carried out with Lipofectamine 2000 (Invitrogen, Carlsbad, CA, USA) according to the manufacturer's instructions. Two days post-transfection, RNA isolation, cell proliferation assay, scratch wound healing assay and matrigel invasion assays were further performed, respectively. The miRNA mimics obtained from GenePharma (Shanghai, China) were as follows: miR-125b mimics sense primer, 5’-UCCCUGAGACCCUAACUUGUGA-3’; miR-125b mimics antisense primer, 5’-ACAAGUUAGGGUCUCAGGGAUU-3’; NC miRNA sense primer, 5’-UUCUCCGAACGUGUCACGUTT-3’; NC miRNA antisense primer, 5’-ACGUGACACGUUCGGAGAATT-3’.

### Total RNA extraction and real-time PCR

Total RNA was extracted using the Trizol reagent (Invitrogen). As for mRNA, first-strand cDNA was synthesized by the PrimeScript RT reagent kit (TaKaRa, Dalian, China) according to the manufacturer's instructions. Real-time PCR was then performed using the SYBR Premix Ex Taq™ II (Takara) in an Applied Biosystems 7500 Fluorescent Quantitative PCR System (Applied Biosystems, Foster City, CA, USA). MiR-125b levels were detected using a TaqMan microRNA kit (Applied Biosystems). In the reverse transcription(RT) step, cDNA is reverse transcribed from total RNA samples using small RNA-specific, stem-loop RT primer from the TaqMan@ small RNA Assays and reagents from the TaqMan@ microRNA reverse transcript kit. The relative amount of miRNA was calculated using ΔΔCt. U6 snRNA was used as an internal control to normalize the experimental results. The Assay ID of miR-125b probe and U6 snRNA probe provided by ABI (USA) were 000449 and 001973. The following primer sequences were used in this work: Has-miR-125b RT primer, 5’-GTCGTATCCAGTGCAGGGTCCGAGGTATTCGCACTGGATACGACTCACAAG-3’; Has-miR-125b sense primer, 5’-GGATTCCCTGAGACCCTAAC-3’; Has-miR-125b antisense primer, 5’-GTGCAGGGTCCGAGGT-3’; U6 RT primer, 5’-AAAATATGGAACGCTTC ACGAATTTGG-3’; U6 sense primer, 5’-CTCGCTT CGGCAGCACATATACT-3’; U6 antisense primer, 5’-ACGCTTCACGAATTTGCGTGTC-3’; ERBB2 sense primer, 5’-TGGCCTGTGCCCACTATAAG-3’; ERBB2 antisense primer, 5’-AGGAGA GGTCAGGTTTCACAC-3’. All amplification processes were normalized by U6 snRNA. The reaction mixtures were incubated at 95°C for 30 sec, followed by 40 amplification cycles of 95°C for 15 sec and 60°C for 60 sec. The comparative Ct method was adopted to quantify relative expression of mRNA. The expression levels of a target gene in a patient were calculated as the ratio of the target expression levels in tumor tissue to those in non-tumorous tissue (T/N).

### Luciferase assay

293TN cell lines were seeded in 96-well plates. Cells were transfected with miR-125b mimics or miRNA-NC. Transfection was performed using Lipofectamine 2000 (Sigma, USA). After 48 h of transfection, Firefly and Renilla luciferase activity was analyzed using the Dual-Luciferase Reporter Assay (Promega, USA), according to the manufacturer's protocol.

### Western blot analysis

Cells in 96-well cell culture plate were rinsed using phosphate-buffered saline, supplemented with protease inhibitors. Then cell lysates were collected and centrifuged at 12,000 rcf and 4°C for 20 minutes. The protein concentrations were measured using the Modified BCA Protein Assay Kit (Vigorous, Beijing). After mixing and boiling with the SDS-PAGE loading buffer, 40 μg of total protein was electrophoresed in 10% SDS-PAGE gels and transferred onto a PVDF (polyvinylidene fluoride) membrane (Merck Millipore, USA). Then the PVDF membrane was blocked in PBST buffer supplemented with 5% skim milk for 2 h at room temperature and incubated with the primary antibodies (HER and GAPDH, CST, USA) overnight at 4°C. After incubation with the consistent horseradish peroxidase-conjugated secondary antibodies (ZSGB-BIO, China), the immunoreactivity was detected using ECL (Merck Millipore, USA).

### Cell proliferation, migration and invasion assays

Following transfection, cell proliferation was assessed using Cell Counting Kit-8 (CCK-8) (Dojindo Inc., Kumamoto, Japan) according to the manufacturer's instructions. HGC-27 and MGC-803 cells were plated in 96-well plates. CCK-8 was added to each well containing culture medium (1:9). The plate was incubated for 2 h at 37°C in a humidified, 5% CO_2_ atmosphere. The plate was measured at a wavelength of 450 nm using a Microplate Reader (Thermo, USA).

For the migration assays, HGC-27 and MGC-803 cells were harvested 48 h post-transfection, and 5×10^4^ cells in 200 μl serum-free RPMI-1640 medium and serum-free DMEM were seeded into the upper transwell chamber, respectively. Cells were visualized at the time of 0, 24, 48, and 72 h using a fluorescence microscope.

A cell invasion assay was performed in chambers consisting of transwell-precoated matrigel membrane filter inserts in 24-well tissue culture plates. The 500 μl culture medium containing 20% FBS in the lower chamber served as the chemoattractant. Cells that had migrated through the filter were stained and counted. The average migration rate was calculated as the increasing radius of the entire cell population over time.

### *In vivo* intratumoral delivery of miR-125b

An intratumoral delivery approach was employed to inspect the effect of miR-125b on the tumor progression *in vivo*. MGC-803 cells (1×10^6^) were injected subcutaneously into nude mice. Once palpable tumors developed at the average volume of 50 mm^3^ (usually on the 10th day), 2 nmol of miR-125b agomir or NC group dissolved in 25 uL of autoclaving sterilized phosphate-buttered saline was delivered intratumorally at 3-day intervals. A total of five injections of mice (n=5) were observed and tumor volume was recorded every 3 days. The tumor volume was calculated as (short diameters^2^ × long diameters)/2. Twenty-six days after administration, all mice were sacrificed and tumor tissues were removed for analysis. All animal care was closely consistent with institutional guidelines. Tumor volumes (mm^3^) at each interval were measured and each tumor in group was weighed to obtain the average. The expression of miR-125b in miR-125b transfection group and NC group was determined by real-time PCR.

### MiRNA microarray analysis

The tumor tissues and adjacent normal tissues of two GC patients were collected for the microarray analysis. Microarray analyses were performed by Bioassay Laboratory of CapitalBio Corporation (Beijing, China). RNA extraction, the purification and dephosphorylation intersected with labeling reaction of total RNA were all performed according to the manufacturer's instructions. In the whole process, labeling spike-in RNA and Hyb spike-in RNA (Agilent Technologies, Inc., Santa Clara, CA, USA) were added and used as quality control. Microarray slides were scanned and microarray images were automatically analyzed using Feature extractionTM software, version 10.7 (Agilent Technology, Inc., Santa Clara, CA, USA). GeneSpring (Agilent Technologies, Inc., Santa Clara, CA, USA) was used to normalize the data and perform variation analysis. Differentially expressed miRNAs were screened out and fold change (FC) > 2 as the criterion for measuring process. Then, the related heat maps were drawn based on the tools reported by Deng W, et al [38]. T-test and ANOVA was employed respectively to analyze the difference of miRNA expressions in two groups and multiple groups (>2).

### Statistical analyses

Statistical analyses were undertaken with SPSS version 17.0 (Statistical Package for the Social Sciences). The data were presented as the mean ± standard deviation (SD). T-test and ANOVA was employed respectively to analyze the difference in two groups and multiple groups (>2). Either the chi-square analysis or the two-tailed Student’s t-test was utilized to appraise the associations of microRNAs in the serum with clinical features, chemotherapeutic response and gastric cancer prognosis. Kaplan–Meier and log-rank test were used to perform survival analysis. Value of *P* < 0.05 was considered statistically significant.

## Abbreviations

DDP: cisplatin
miRNAs: microRNAs
NC: negative control
real-time PCR: real-time polymerase chain reaction
PFS: progression free survival
OS: overall survival
DMEM: Dulbecco’s Modified Eagle’s Medium
FBS: fetal bovine serum
CCK-8: Cell Counting Kit-8.
