# Correction: The functional mechanism of miR-125b in gastric cancer and its effect on the chemosensitivity of cisplatin

**DOI:** 10.18632/oncotarget.27405

**Published:** 2020-01-28

**Authors:** Xinyue Zhang, Jie Yao, Kai Guo, Hu Huang, Siyuan Huai, Rui Ye, Baolong Niu, Tiannan Ji, Weidong Han, Jianxiong Li

**Affiliations:** ^1^ Department of Radiotherapy, Chinese PLA General Hospital, Beijing 100853, P.R. China; ^2^ Center for Evidence-Based and Translational Medicine, Zhongnan Hospital of Wuhan University, Wuhan 430071, P.R. China; ^3^ Department of Gastroenterology, The 161th Hospital of PLA, Wuhan 430010, P.R. China; ^4^ Department of Oncology, The 161th Hospital of PLA, Wuhan 430010, P.R. China; ^5^ Department of Oncology, Beidaihe Sanatorium of Beijing Military Command, Qinhuangdao 066100, P.R. China; ^6^ Department of Molecular Biology, Institute of Basic Medicine, School of Life Sciences, Chinese PLA General Hospital, Beijing 100853, P.R. China; ^7^ Department of Radiotherapy, Hainan Branch of Chinese PLA General Hospital, Sanya 572000, P.R. China

**This article has been corrected:** Due to errors in figure processing, the image in miR-125b mimics group at 0h is exactly the same as the NC group in Figure 5B. The corrected Figure 5B is attached. The authors declare that these corrections do not change the results or conclusions of this paper.

Original article: Oncotarget. 2018; 9:2105–2119. 2105-2119. https://doi.org/10.18632/oncotarget.23249

**Figure 5:**
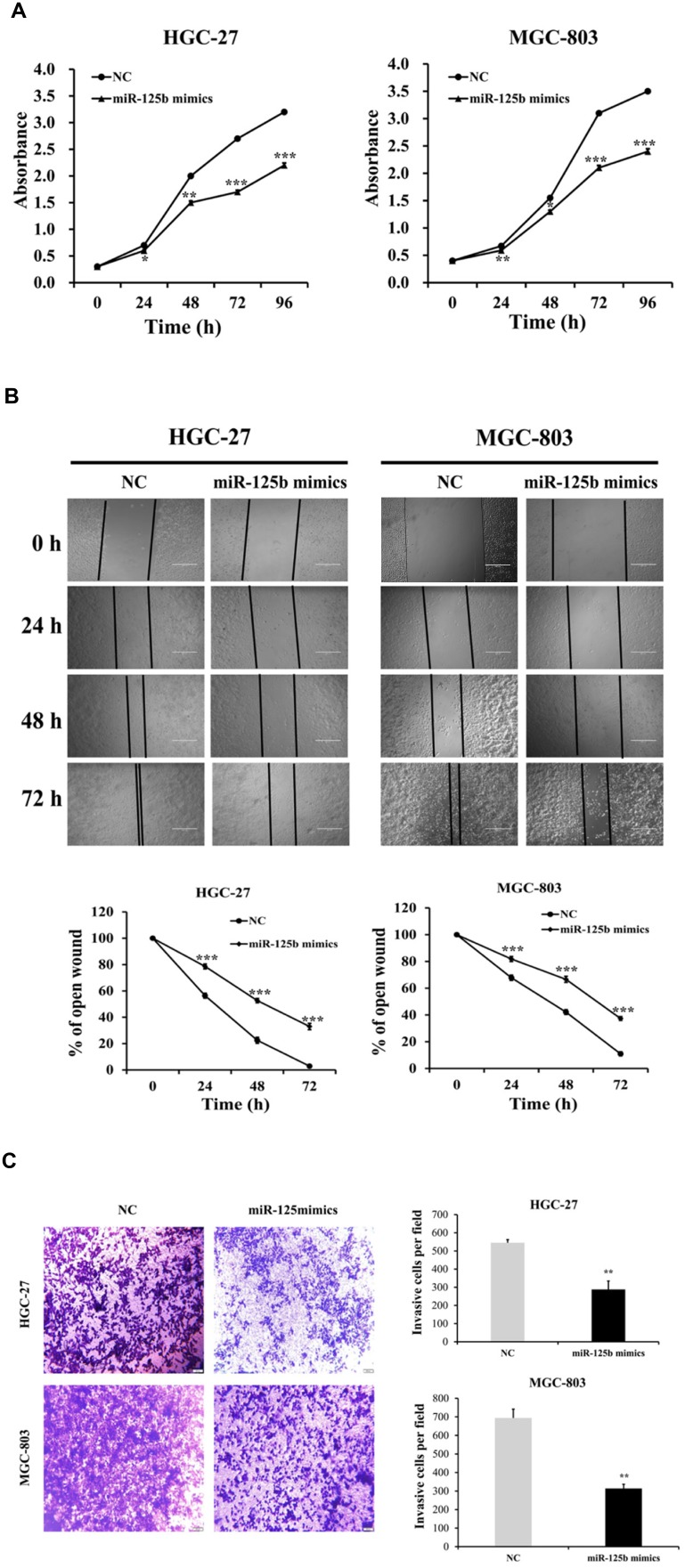
miR-125b expression levels inhibited the proliferation, migration and invasion abilities of HGC-27 and MGC-803 cells.

(**A**) In miR-125b overexpression experiment, the data indicated that both the proliferation of HGC-27 and MGC-803 cells in miR-125b mimics groups were evidently retarded compared with control group. (**B**) The wound-healing assay showed that HGC-27 and MGC-803 cells migration in miR-125b mimics groups were inhibited compared with the control groups. (**C**) The matrigel invasion assay also displayed the suppression in miR-125b mimics groups compared with the control groups. Error bars symbolized standard deviation were obtained from three independent experiments and all the data were shown as mean ± SD. All scale bars in Figure B and Figure C are 200 μm and 500 μm, respectively. NC, negative control; h, hour; *, *P* < 0.05; **, *P* < 0.01; ***, *P* < 0.001.

